# Gene Expression Analysis Identifies Novel Targets for Cervical Cancer Therapy

**DOI:** 10.3389/fimmu.2018.02102

**Published:** 2018-09-19

**Authors:** Jason Roszik, Kari L. Ring, Khalida M. Wani, Alexander J. Lazar, Anna V. Yemelyanova, Pamela T. Soliman, Michael Frumovitz, Amir A. Jazaeri

**Affiliations:** ^1^Department of Genomic Medicine, The University of Texas MD Anderson Cancer Center, Houston, TX, United States; ^2^Department of Melanoma Medical Oncology, The University of Texas MD Anderson Cancer Center, Houston, TX, United States; ^3^Division of Gynecologic Oncology, Department of Obstetrics and Gynecology, University of Virginia Health System, Charlottesville, VA, United States; ^4^Department of Translational Molecular Pathology, The University of Texas MD Anderson Cancer Center, Houston, TX, United States; ^5^Department of Pathology, The University of Texas MD Anderson Cancer Center, Houston, TX, United States; ^6^Department of Pathology, The University of Alabama at Birmingham, Birmingham, AL, United States; ^7^Department of Gynecologic Oncology and Reproductive Medicine, The University of Texas MD Anderson Cancer Center, Houston, TX, United States

**Keywords:** cervical, cancer, retrospective analysis, gene expression profile, immunohistochemistry, combination therapies

## Abstract

Although there has been significant progress in prevention and treatment of cervical cancer, this malignancy is still a leading cause of cancer death for women. Anti-angiogenesis and immunotherapy approaches have been providing survival benefits, however, response rates and durability of response need to be improved. There is a clear need for combination therapies that increase effectiveness of these agents and further improve patient outcome. Previous studies have largely focused on gene expression and molecular pathways in untreated cervix cancer. The goal of this study was to evaluate cancer-specific molecular pathways and their correlation with tumor immune profile in recurrent cervical cancer. Tumor and adjacent normal tissues were used to identify potential combination therapy targets. We found that DNA damage repair pathway genes were significantly overexpressed in the tumor. Based on our results and other recent investigations, we suggest that combination immune checkpoint and PARP inhibitor therapy is a high priority consideration for patients with recurrent, previously treated cervical cancer. We also show that multiple epithelial-mesenchymal transition-related genes, including MAP2K4, ID2, JAK1, FGF2, PIK3R1, AKT3, FGF13, and STAT3 may be potential targets. Interestingly, high-throughput analysis of Cancer Genome Atlas data identified distinct targets, including Fatty acid synthase FASN and Matrix Metallopeptidase 1 MMP1 as novel, promising combination therapy partners.

## Introduction

Despite effective screening and preventative vaccines, there will be an estimated 13,240 new cases of cervical cancer and 4,170 deaths estimated in 2018 in the United States, with cervical cancer accounting for the second leading cause of cancer death for women age 20 to 39 years (1). Unfortunately, women who present with advanced stage and metastatic disease have a poor prognosis and limited therapeutic options. Response rates to current therapies range from 35 to 50% with a median survival of less than 2 years (2, 3). Biologic therapy targeting the vascular endothelial growth factor (VEGF) has shown the most recent success in the treatment of cervical cancer and is now used in combination with chemotherapy as the standard of care in the treatment of recurrent and metastatic disease. While anti-angiogenesis therapies have shown an incremental improvement in survival, there remains a high clinical demand for novel treatment strategies in this disease site. At this time, there are no other targeted therapies approved in the treatment of cervical cancer according to clinicaltrials.gov.

Immunotherapy presents an additional rational approach for the treatment of cervical cancer given the molecular underpinnings of this human papilloma virus (HPV) related disease. Impaired local cellular immunity results in persistent infection with high risk HPV and expression of viral oncoproteins E6 and E7. Expression of these oncoproteins in turn leads to downstream genomic instability through interactions with the well described tumor suppressor genes p53 and retinoblastoma (pRb). The loss of cell cycle regulation allows for an increased mutational burden and malignant transformation from cervical intraepithelial neoplasia to invasive carcinoma (4). An increased mutational burden is a source of targetable neoantigens that can be detected in most cervix tumors (5). Cervical cancer, as well as other HPV related diseases, also present a unique viral antigen for T-cells to identify tumor cells from self and serve as ideal candidates for immunotherapy from a biologic standpoint. In contrast, previous studies have also demonstrated that immune checkpoint pathways, including PD-1 /PD-L1 are activated during chronic viral infections to combat T-cell responses to viral antigens. Yang and colleagues evaluated this in cervical intraepithelial neoplasia and showed that upregulation of the PD-1/PD-L1 pathway was associated with HPV positivity and progression of precancerous lesions (6). In addition, diffuse PD-L1 expression has been associated with worse disease specific survival in this patient population (7).

Multiple trials evaluating various immunotherapy strategies are currently underway in both the upfront and recurrent setting, however immune checkpoint blockade has been the most widely studied in cervical cancer to date (clinicaltrials.gov) and the majority of trials have evaluated this strategy in the single agent setting. While initial studies have shown activity of checkpoint blockade in cervical cancer, response rates thus far are disappointing and range from 17 to 27% (8, 9). Despite these low response rates pembrolizumab recently gained FDA approval for patients with recurrent or metastatic cervical cancer with disease progression on or after chemotherapy whose tumors express PD-L1. The current therapeutic landscape highlights the need for: (a) rationale combination therapies with the potential to provide improved responses, and (b) identification of molecularly defined subgroups who may benefit from immunotherapy. Our objective was to identify immune related as well as other potentially targetable cancer pathways in recurrent cervical cancer in an effort to identify rational combination therapies that should be prioritized in developmental therapeutics for cervical cancer (10).

## Results

### Clinical characteristics

Twenty-eight patients were treated for recurrent cervical cancer with a pelvic exenteration at MD Anderson Cancer Center from 1994 to 2004. We focused our investigation on this population, because it represents the population with the greatest unmet need in this disease (i.e., patients with recurrent disease following primary treatment with surgery and/or chemoradiation). The majority of patients initially presented with squamous histology (*n* = 19, 67.9%) and locally advanced disease (*n* = 17, 60.7%) Nineteen patients were treated with primary chemoradiation (67.9%), 2 were treated with a radical hysterectomy (7.1%), and 7 (25.0%) were treated with both a hysterectomy and chemoradiation. Seven patients (25.0%) were alive with no disease as of last follow-up and 16 (57.1%) had died as a result of recurrent disease (Table 1).

**Table 1:** Clinical characteristics of patients.

**Clinical characteristics *N* = 28**	**N (%)**
**RACE**	
White African American Hispanic Asian	17 (60.7) 2 (7.1) 8 (28.6) 1 (3.6)
**STAGE**	
IA2 IB1 IB2 IIA IIB IIIA IIIB IVA Unknown	1 (3.6) 8 (28.6) 5 (17.8) 0 (0) 6 (21.4) 1 (3.6) 4 (14.3) 1 (3.6) 2 (7.1)
**GRADE**	
1 2 3 Unknown	3 (10.7) 11 (39.3) 11 (39.3) 3 (10.7)
**HISTOLOGY**	
Squamous cell carcinoma Adenocarcinoma	19 (67.9) 9 (32.1)
**PRIMARY TREATMENT**	
Radical hysterectomy Primary chemoradiation Hysterectomy and Chemoradiation	2 (7.1) 19 (67.9) 7 (25.0)
**DISEASE STATUS AT LAST FOLLOW-UP**	
Alive, No evidence of disease Alive with disease Dead with disease Dead of unknown cause	7 (25.0) 1 (3.6) 16 (57.1) 4 (14.3)

### Nanostring expression analysis identifies immune alterations in cervix tumors

**Figure 1:**
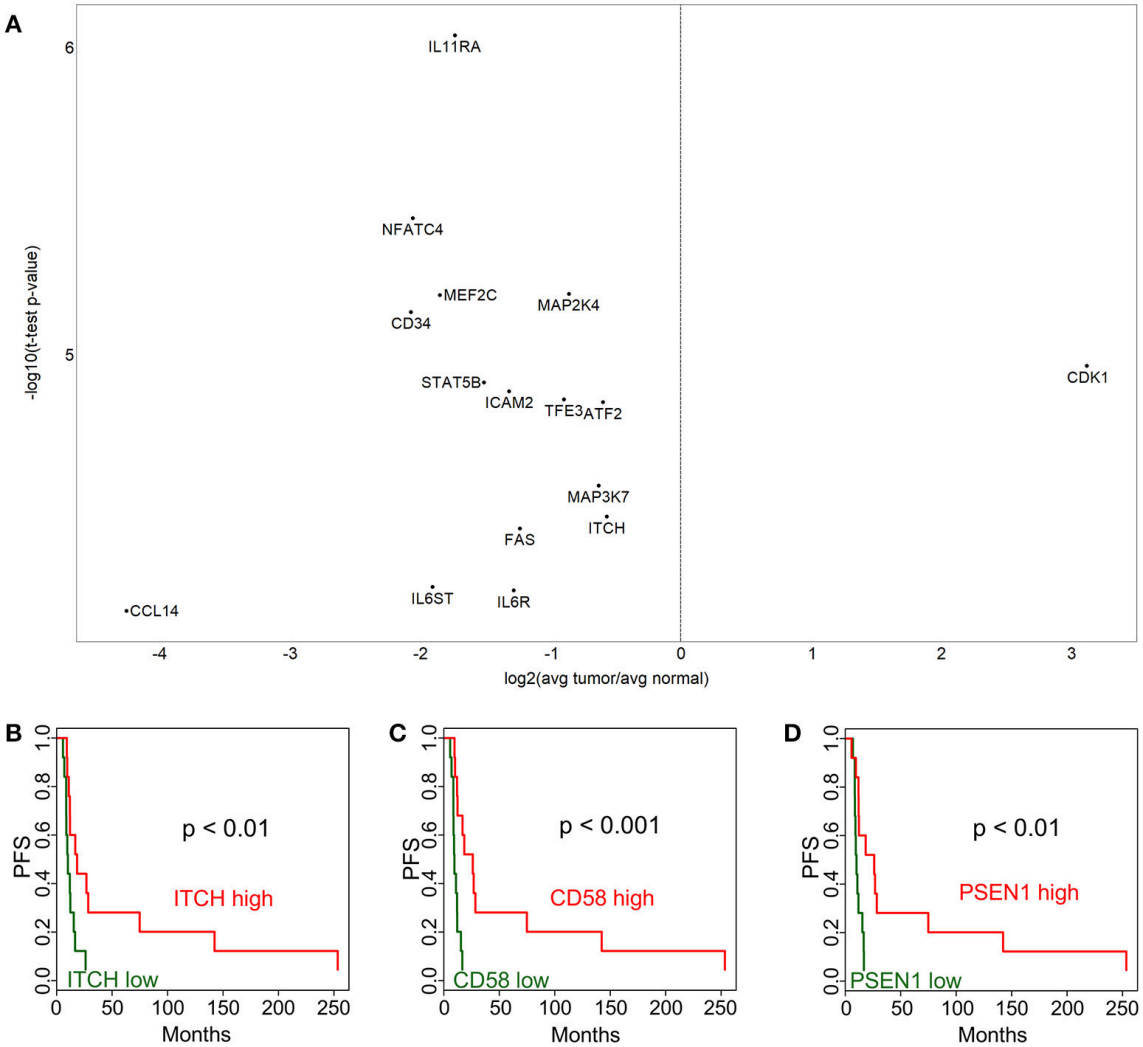
Immune-related gene expression alterations in cervical cancer. Over- and under-expressed immune-related genes in cervix tumors compared to adjacent normal tissue are shown in **(A)**. Association between progression-free survival (PFS) and gene expression is displayed for ITCH **(B)**, CD58 **(C)**, and PSEN1 **(D)**. High expression (above median) is depicted with red color, while low expression (below median) is green.

We have performed a NanoString expression analysis using the Cancer Immune genes code set (*n* = 730 genes) to compare tumor and adjacent normal tissue in the patient cohort described in Table 1. Figure 1A shows the differentially expressed genes, after Bonferroni correction for multiple tests. Interestingly, only cyclin-dependent kinase 1 (CDK1) showed overexpression in the tumor. Genes that were expressed at a lower level in the tumor included IL11RA, NFATC4, MEF2C, MAP2K4, MAP3K7, CD34, STAT5B, ICAM2, TFE3, ATF2, FAS, ITCH, CCL14, IL6ST, and IL6R. Low expression of the ITCH gene was also associated with significantly (*p* < 0.01) shorter progression-free survival (PFS) (Figure 1B). We next sought to identify immune related genes expressed in the tumor that correlated with survival. We identified CD58 (lymphocyte function-associated antigen 3 - LFA-3) cell adhesion molecule and macrophage marker, and PSEN1 (presenelin 1), a chemoresistance-associated gene, were significantly associated with improved progression free survival (PFS; Figures 1C, D). The *p*-values from Kaplan-Meier analyses of all genes can be found in Supplementary Table 1.

### Cancer pathways show more dominant alterations compared to immune genes

**Figure 2:**
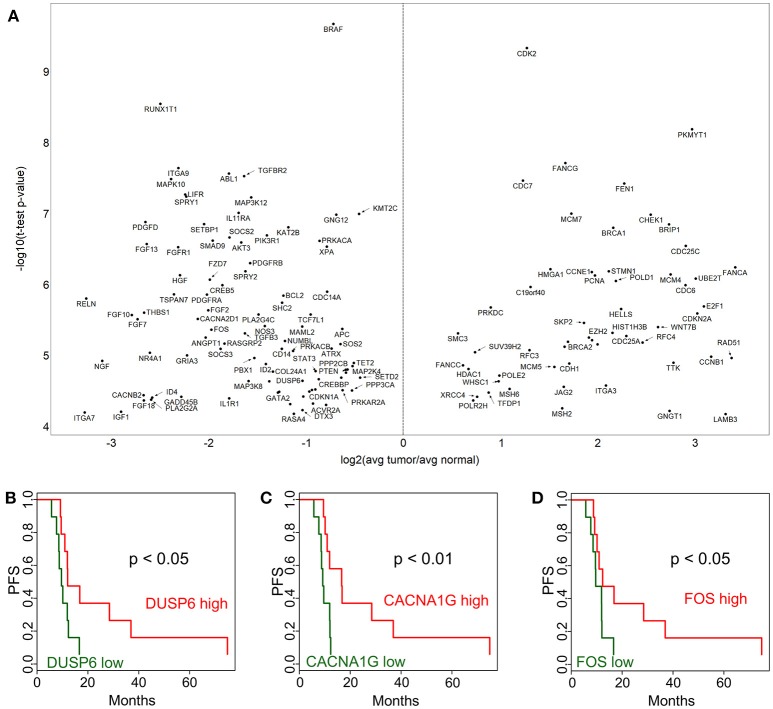
Cancer pathway-associated genes show diverse alterations in cervical cancer. Cervix tumor and adjacent normal tissue expression comparisons are shown in **(A)** for genes in the NanoString Cancer Pathways code set. Progression-free survival (PFS) associations for selected genes in the NanoString panel, DUSP6 **(B)**, CACNA1G **(C)**, and FOS **(D)** are displayed comparing high (above median) and low (below median).

In order to identify cancer-related pathways that may serve as potential targets for therapeutic intervention, we performed a NanoString study using the Cancer Pathways code set which is composed of 730 genes. In this analysis we identified 423 genes that were differentially expressed at *p* < 0.05 level. Out of these, 148 were significant after Bonferroni correction (Figure 2A). A notable observation was the significantly higher expression of DNA damage repair pathway genes, in particular those involved in homologous recombination and mismatch repair pathways) in tumor tissues (Figure 2A, e.g., BRCA1, BRCA2, BRIP1, FANCA, FANCG, FANCC, RAD51, XRCC4, MSH2, MSH6, MCM7, MCM4, PCNA). Our findings are especially intriguing, because it was recently shown that HPV E6 and E7 oncogenes increase the abundance of HR proteins and enhance their ability to form DNA repair foci. However, ironically, E6 and E7 interfere with the ability of the HR pathway to complete double-strand break (DSB) repair, resulting in homologous recombination deficiency (HRD) (11).

In addition, Ingenuity Pathway Analysis revealed that 25 out of these are in the “Regulation of the Epithelial-Mesenchymal Transition Pathway” (MAP2K4, ID2, JAK1, FGF2, PIK3R1, SOS2, FGF13, TGFBR2, BRAF, FGF10, FGF18, HGF, WNT7B, AKT3, FGF7, PDGFRB, JAG2, FGFR1, STAT3, TCF7L1, APC, CDH1, TGFB3, PDGFD, and FZD7). This pathway appeared to be suppressed in tumor cell compared to adjacent normal tissue. A few selected PFS associations are shown for DUSP6 (Figure 2B), CACNA1G (Figure 2C), and FOS (Figure 2D). The *p*-values from Kaplan-Meier analyses are provided for all genes in Supplementary Table 2. These expression and survival differences clearly show that multiple oncogenic pathways are active.

### Immunohistochemistry analysis indicates PFS associations

**Figure 3:**
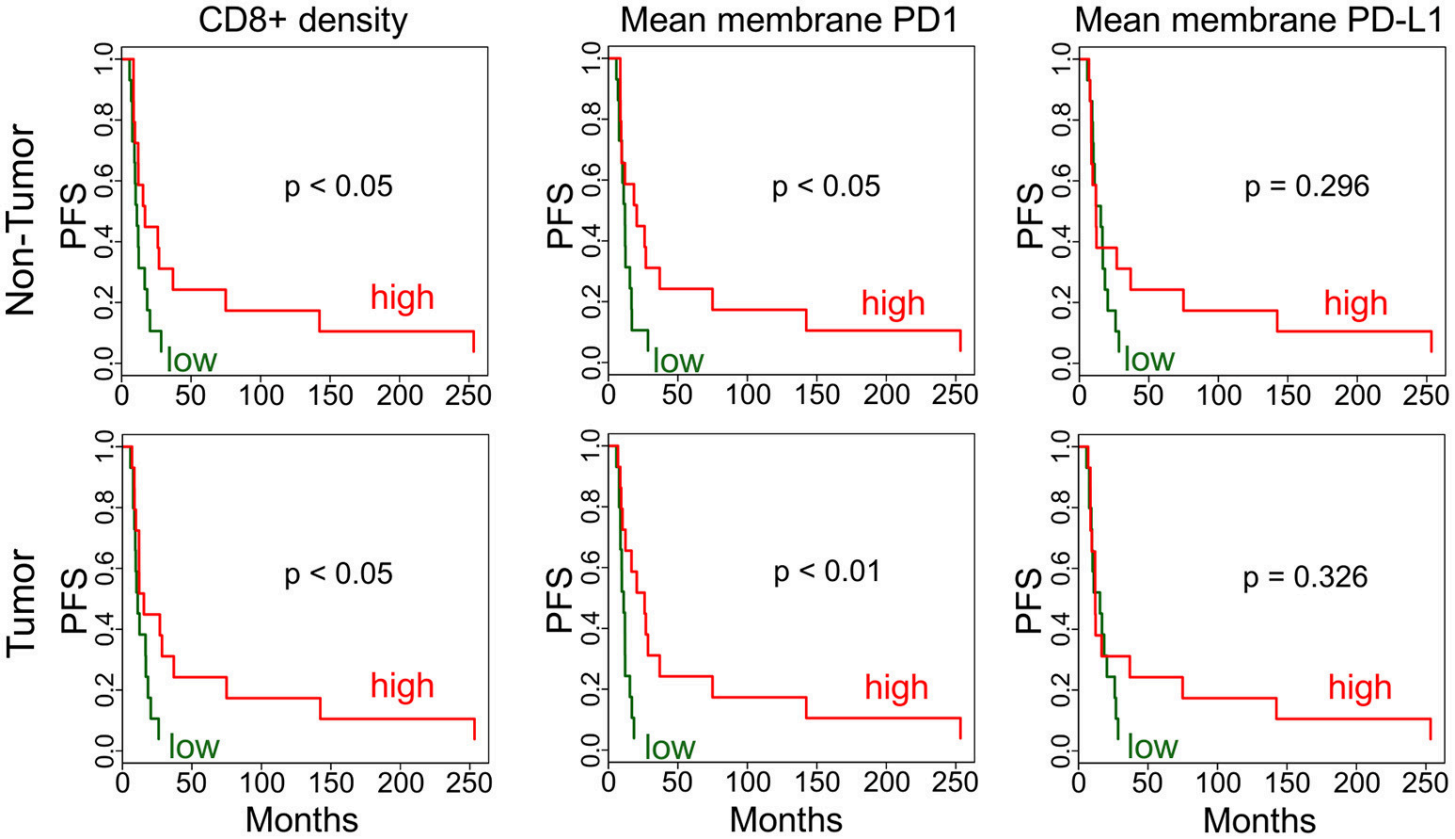
Immunohistochemistry analysis of tumors and adjacent non-tumors. Kaplan-Meier plots are applied to analyze PFS associations in non-tumor and tumor for CD8+ phenotype density (counts/mm^2^) and mean membrane PD1 and PD-L1 expression. High (red) is the above median density/expression group, low (green) is below median.

In addition to gene expression level comparisons, we also analyzed PFS associations with protein level by immunohistochemistry (IHC). We found that higher CD8+ density and mean membrane PD1 level were associated with better survival (*p* < 0.05) both in adjacent non-tumor and in tumor (Figure 3). FoxP3+ phenotype density did not show a statistically significant association with PFS. The PD-L1 expression PFS-association was also not significant, possibly due to the high variation in expression in tumors.

### Immunologic features of cervical cancer are associated with targetable molecular pathways

**Figure 4:**
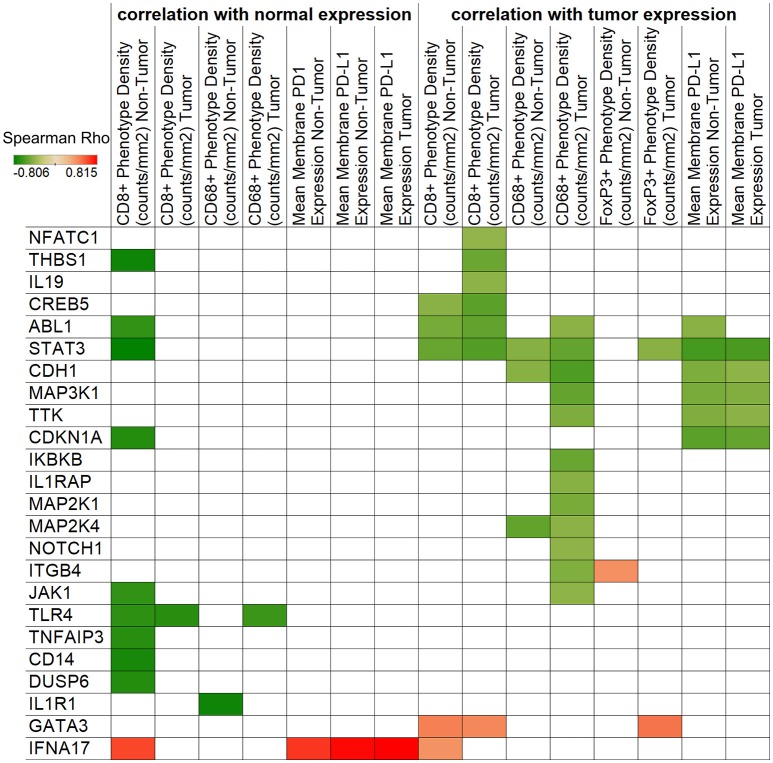
Correlation of IHC and NanoString expressions. Spearman's rank correlation coefficients are represented by color for NanoString genes and IHC densities/expressions. The left side of the figure shows correlations with normal tissue gene expression, while the right side displays tumor expression associations. Only significant (*p* < 0.05) correlations are shown.

We next sought to correlate the immunologic features on cervical tumor microenvironment with gene expression levels to gain additional insight into the potential interactions between the immune and cancer pathways. We performed an unbiased correlation analysis of tumor immune phenotype (as revealed by IHC) and gene expression (using Nanostring) (Figure 4). One of the notable observations in this analysis was the negative correlation between STAT3 expression with CD8+ phenotype density. This is important because STAT3 can be over-expressed and constitutively-activated in cervical cancer (12), and decreasing its expression may help to increase CD8+ cell infiltration to the tumor. Another important observation is that macrophage marker CD68+ phenotype density was found to be associated with multiple markers in the tumor including STAT3, ABL1, CDH1, MAK3K1, MAP2K1, MAP2K4, TTK, IKBKB, IL1RAP, NOTCH1, ITGB4, and JAK1.

### TCGA analysis reveals potential combination therapies

**Figure 5:**
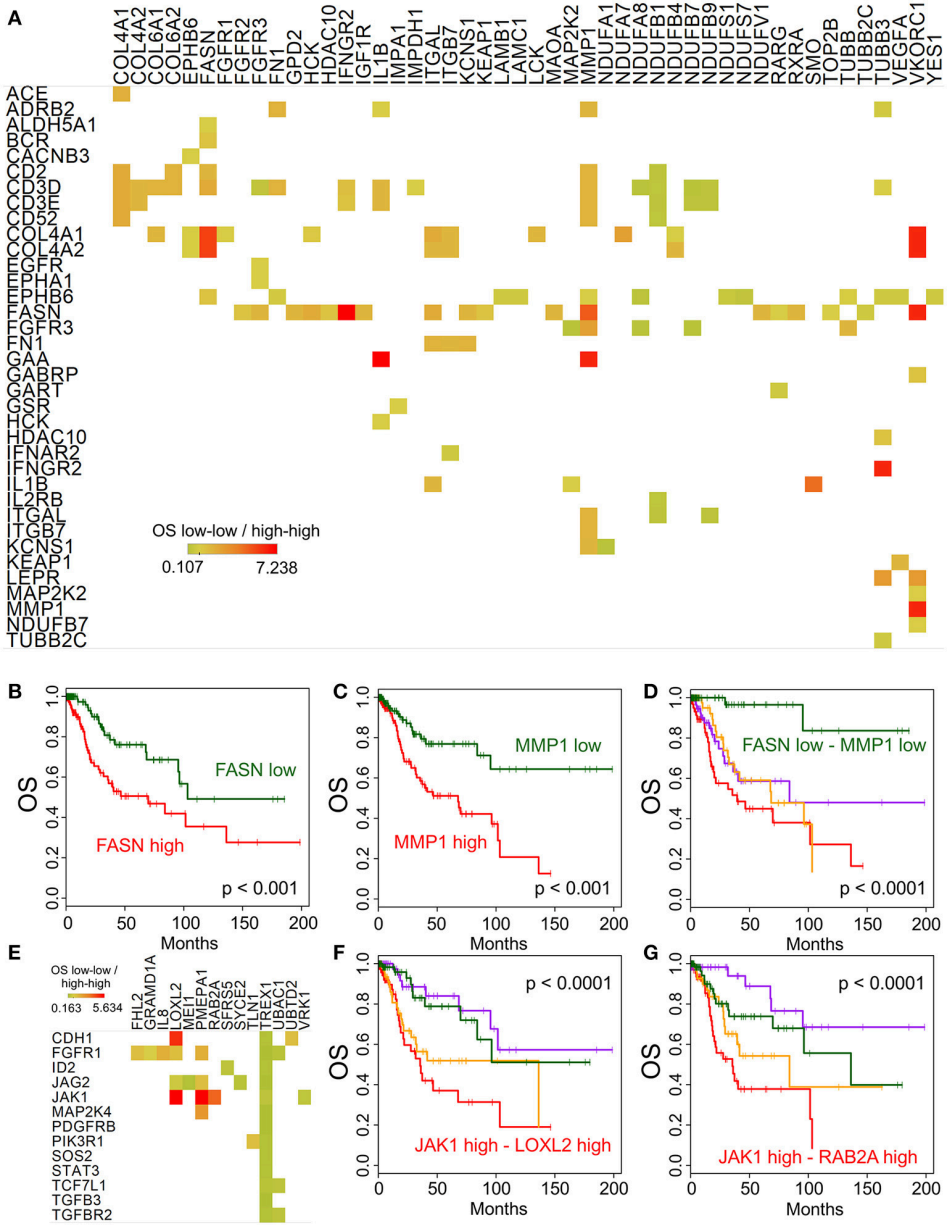
Potential targets for combination therapies. Median overall survival ratio of low-low (both below median) and high-high (both above median) expression of gene pairs is depicted as shown by the color legend **(A)**, only *p* < 0.0001 associations where median expression >5 TPM). Kaplan-Meier plots show the difference between high and low expression for FASN **(B)** and MMP1 **(C)** alone, and in combination **(D)**. **(E)** Displays the overall survival ratio analysis results for the EMT genes (shown in rows) identified by our Ingenuity Pathway Analysis. The JAK1 - LOXL2 **(F)** and JAK1 - RAB2A **(G)** combination survival analyses are shown as examples.

While the main focus of our study was the immune and molecular phenotype of recurrent cervical cancer, the availability of the Cancer Genome Atlas (TCGA) cervix cancer data provided an opportunity to correlate gene expression and survival in untreated cervical cancer samples (used in TCGA). We performed this analysis using the high-throughput architecture which we developed earlier (13). The association of overall survival and gene expression was determined for gene pairs using the following four groups: (1-high-high group): the expression of both genes is above median expression; (2-high-low group): the expression of the first gene is above while the second gene is below median expression; (3-low-high group) the expression of the first gene is below and the expression of the second is above median expression; and (4-low-low group) the expression of both genes is below median expression levels. Figure 5A shows the gene pairs where high expression of both genes of a potential combination is associated with shorter or longer median survival than low expression of the same genes. One of the most interesting combination we identified is Fatty acid synthase FASN and Matrix Metallopeptidase 1 MMP1. Low expression of either of these genes is associated with significantly longer overall survival (Figures 5B,C), however, when both genes have low expression (Figure 5D), median survival is much more improved (*p* < 0.0001). We have also performed a similar analysis focusing on the EMT genes identified above. Figure 5E shows the gene pairs with significant overall survival association, and the JAK1 - LOXL2 (Figure 5F) and JAK1 - RAB2A (Figure 5G) combinations are also shown using Kaplan-Meier plots.

## Discussion

Immunotherapy can take many forms including therapeutic vaccines, adoptive transfer of autologous tumor-infiltrating T lymphocytes (TILs), chimeric antigen receptor (CAR)-engineered T-cells, and immune checkpoint blockade. Optimal immunotherapies as well as targeted therapies usually require target overexpression in the tumor compared to normal tissues (14). To identify such targets, we performed gene expression and IHC analyses of tumor and adjacent normal tissues of 28 cervical cancer patients.

Our NanoString Cancer Immune expression analysis identified multiple immune alterations in cervix tumors, but most of the genes with significantly altered expression were under-expressed in the tumor, making them unattractive targets. However, CDK1 was over-expressed, and may be a potential combination therapy target. In addition to this gene, our NanoString Cancer Pathways analysis provided a number of promising target candidates. Several DNA damage repair pathway genes were overexpressed in the tumor. In particular there was an overabundance of genes in the BRCA-Fanconi Anemia and homologous recombination pathways. These findings along with a previous report that HPV E6 and E7 oncogenes increase the abundance of HR while producing functional homologous recombination deficiency (HRD) (11), support the clinical investigation of poly (ADP-ribose) polymerase inhibitors (PARPi) alone or in combination with other therapies in recurrent cervical cancer. In fact a phase I study of paclitaxel, cisplatin, and the PARPi veliparib in the treatment of persistent or recurrent carcinoma of the cervix found this combination safe and feasible (15). Another study of rucaparib and bevacizumab combination in patients with recurrent cervical cancer is currently ongoing (NCT03476798). Considering both the immune and gene expression profiling data provided by our study we feel that combination immune checkpoint and PARPi therapy is a high priority consideration in patients with recurrent previously treated cervical cancer.

Furthermore, epithelial-mesenchymal transition (EMT) was one of the top pathways associated with these differentially expressed genes. Although EMT has been shown to be targetable in cervical cancer, the regulation of EMT is not well known (16). Out of the 25 genes identified in our study, multiple have been implicated in cancer and may be targetable, including MAP2K4 in prostate cancer (17), ID2 in glioma (18), JAK1 in multiple cancer types (19), FGF2 to address resistance to anti-VEGF therapy (20), PIK3R1 and AKT3 using PI3K/AKT/mTOR inhibitors (21), FGF13 which mediates resistance to platinum therapy in cervical cancer (22), and STAT3 which has also been proposed as a target in cervical cancer (23).

Our IHC analyses clearly indicate immune activity in cervix cancer, however, cytotoxic T cell density was found to be lower in tumors compared to a relatively high density in adjacent normal tissues. FoxP3+ regulatory T cells (Tregs) may be partially responsible for an immune-suppressive tumor microenvironment, however, our results indicate that Tregs are also excluded from tumors to adjacent areas. CD68+ macrophages were present in high quantities in both tumor and adjacent non-tumor tissues. We also showed that CD68+ phenotype density was inversely associated with NOTCH1 expression. Notch1-activating agents have been proposed earlier (24), and based on our results this may be considered together with targeting macrophages in the tumor. We also identified some PD-L1 protein expression, which may be a useful marker for a subset of patients for immune checkpoint inhibitor therapy (25). Interestingly, PD-L1 gene expression may also be associated with higher neoantigen burden and expression of HPV master regulators (5).

In addition, our TCGA-based high-throughput combination therapy prediction identified Fatty acid synthase (FASN) and Matrix Metallopeptidase 1 (MMP1) as a promising target candidate pair. FASN inhibitors have shown promising results for therapy of breast cancer (26) and orthotopic tongue oral squamous cell carcinoma (27). MMP1 is overexpressed in cervical cancer, and knockdown of MMP1 reduced the proliferation and migration of cervical cancer cells, while expression of epithelial marker E-cadherin increased and expression of mesenchymal marker Vimentin decreased (28). These and our results suggest that a combined FASN and MMP1 inhibition may be beneficial for cervical cancer patients.

We would like to acknowledge some of the limitations of this study. This study was retrospective and performed on a small but relevant patient population in that all patients had recurrent disease and had received prior radiation representing the at need population. Despite this limitation, these findings present an opportunity to rationally approach future combination immunotherapy trials in the treatment of recurrent cervical cancer.

## Methods

### Patient sample preparations

Following institutional review board approval (PA15-0286), patients were identified retrospectively through a departmental database (MDA 2008-0095). All women with a diagnosis of squamous cell carcinoma or adenocarcinoma of the cervix who underwent a pelvic exenteration procedure at The University of Texas MD Anderson Cancer Center from 1994 to 2004 were included. Clinical and pathologic data were abstracted from the medical record. Formalin-fixed, paraffin-embedded tumor samples were identified and specimens were reviewed for pathologic diagnosis and dissected if necessary to ensure that ≥90% of the sample represented tumor. The normal tissue was directly adjacent to the tumor on the same slide.

### Nanostring analyses

NanoString was performed using the nCounter® PanCancer Immune Profiling panel (XT–CSO-HIP1-12) composed of 770 immune response genes and the nCounter® PanCancer pathways panel (XT–CSO-PATH1-12) comprised of 770 genes from 13 canonical cancer associated pathways (NanoString Technologies). RNA was extracted using the Highpure miRNA isolation kit (Roche) from FFPE blocks, following initial confirmation of tumor presence and content by two pathologists by H&E. For gene expression studies, 1 μg of RNA was used as per manufacturer's instructions (NanoString Technologies). All samples included in this study passed a quality check before the NanoString analysis. Raw NanoString results were normalized using standard NanoString housekeeping genes before comparing tumor-normal pairs. We performed a “Core Analysis” with the significantly differently expressed genes (after Bonferroni correction) in the Cancer Immune and separately with the significant results in the Cancer Pathways panel.

### Immunohistochemistry

We performed further survival and expression correlation analyses with our previously published immunohistochemistry data described in (29).

### Statistical analyses

To compare tumors and normals we used two-tailed paired Student's *t*-tests. Statistically significant differences were noted when *p* < 0.05. In expression analyses we used Bonferroni correction to adjust the 0.05 threshold of significance for comparing expression level of 730 genes.

### Data visualization

We used the Tableau Desktop business intelligence tool to prepare the figures. Kaplan-Meier plots were made using the “survival” R package.

## Ethics statement

This study was carried out in accordance with the approval of MD Anderson Cancer Center IRB with a waiver of informed consent due to the retrospective and minimal risk nature of this study.

## Author contributions

AJ: Study concept and design; JR, KR, KW, AL, AY, PS, MF, and AJ: Acquisition, analysis, or interpretation of data; JR, KR, KW, AL, AY, PS, MF, and AJ: Preparation, review, or approval of the manuscript.

### Conflict of interest statement

The authors declare that the research was conducted in the absence of any commercial or financial relationships that could be construed as a potential conflict of interest.
